# Tc-99m-ECD SPECT as the measure for therapeutic response in patients with cobalamin deficiency: Two case reports

**DOI:** 10.1097/MD.0000000000004851

**Published:** 2016-09-30

**Authors:** Min-Chien Tu, Chung-Ping Lo, Ching-Yuan Chen

**Affiliations:** a Department of Neurology; b Department of Radiology; c Department of Nuclear Medicine, Taichung Tzu Chi Hospital, Buddhist Tzu Chi Medical Foundation, Taichung; d School of Medicine, Tzu Chi University, Hualien; e Graduate Institute of Medical Imaging and Radiological Sciences, Central Taiwan University of Science and Technology, Taichung, Taiwan.

**Keywords:** cobalamin, cognition, SPECT, vitamin B12

## Abstract

**Background:**

Cobalamin (Cbl) is an essential vitamin for human health. While an increasing body of evidence supports the negative impact of Cbl deficiency on cognition, the causality has yet to be determined, and the reported therapeutic responses after Cbl supplement therapy have been inconsistent. Besides, few reports have described neuroimaging characteristics associated with the therapeutic response.

**Methods:**

To describe and compare technetium-99m ethyl cysteinate dimer single-photon emission computed tomography (Tc-99m-ECD SPECT) findings in 2 patients with Cbl deficiency with distinct therapeutic responses.

**Results:**

*Case 1* scored 12/30 in the mini-mental state examination (MMSE) and 34/100 in the cognitive abilities screening instrument (CASI). Profound deficits in mental manipulation, drawing, short-term/long-term memory, and verbal fluency were noted. *Case 2* scored 24/30 in the MMSE and 78/100 in the CASI, mainly due to impaired mental manipulation, abstract thinking, and borderline performance in short-term memory and verbal fluency. While both cases showed widespread hypoperfusion within bilateral frontotemporal regions and thalamus on Tc-99m-ECD SPECT, *Case 2* demonstrated relatively preserved radio-uptake in the frontal regions, especially the anterior cingulate cortex (ACC) and prefrontal cortex (PFC), consistent with the better therapeutic response (*Case 1*: 12/30 to 11/30 in the MMSE; *Case 2*: 24/30 to 28/30 in the MMSE).

**Conclusion:**

Given that the ACC integrates the limbic system and frontosubcortical circuits and the PFC governs executive function, the extent and severity of hypofrontality may be responsible for the worse prognosis. Our Tc-99m-ECD SPECT observations revealed that the negative impact on cerebral metabolic tone is relevant to the severity of Cbl deficiency, and the functional integrity of the ACC and PFC is highly associated with the preservation of global cognitive function in our cases with Cbl deficiency.

## 1 Introduction

Cobalamin (Cbl) is an essential vitamin for human health.^[1]^ Due to its pivotal role in both deoxyribonucleic acid and fatty acid synthesis, the neurological presentations of Cbl deficiency are diverse and may present with variable severity.^[1]^ While an increasing body of evidence supports the negative impact of Cbl deficiency on cognition,^[2,3]^ the causality has yet to be determined, and the reported therapeutic responses after Cbl supplement therapy have been inconsistent.^[2]^ Some studies have reported disease severity^[2]^ and baseline levels of methylmalonic acid^[4]^ to be predictors of the prognosis. However, few reports have described neuroimaging characteristics associated with the therapeutic response. Therefore, the aim of this study was to describe and compare technetium-99m ethyl cysteinate dimer single-photon emission computed tomography (Tc-99m-ECD SPECT) findings in 2 patients with Cbl deficiency with distinct therapeutic responses. Institutional review board approval was waived, as this is a retrospective case report with no identifying patient information presented. Written informed consent was obtained from the patients for the publication of this case report. A copy of the written consents is available for review by the editor of this journal.

## 2 Case reports

**Figure 1:**
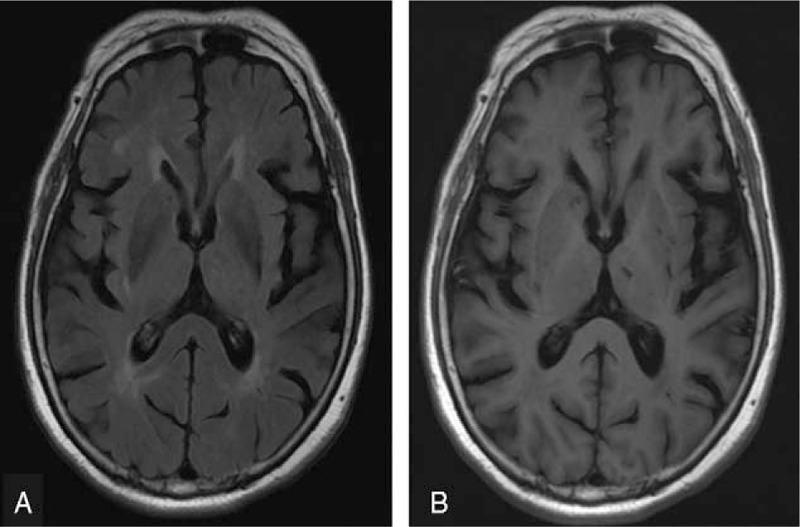
Brain magnetic resonance imaging of Case 1 showing small periventricular caps and subtle senile atrophy, which were both disproportionate to her cognitive deficits. (A) T2 fluid-attenuated inversion recovery image, axial view. (B) T1-weighted image, axial view.

**Figure 2:**
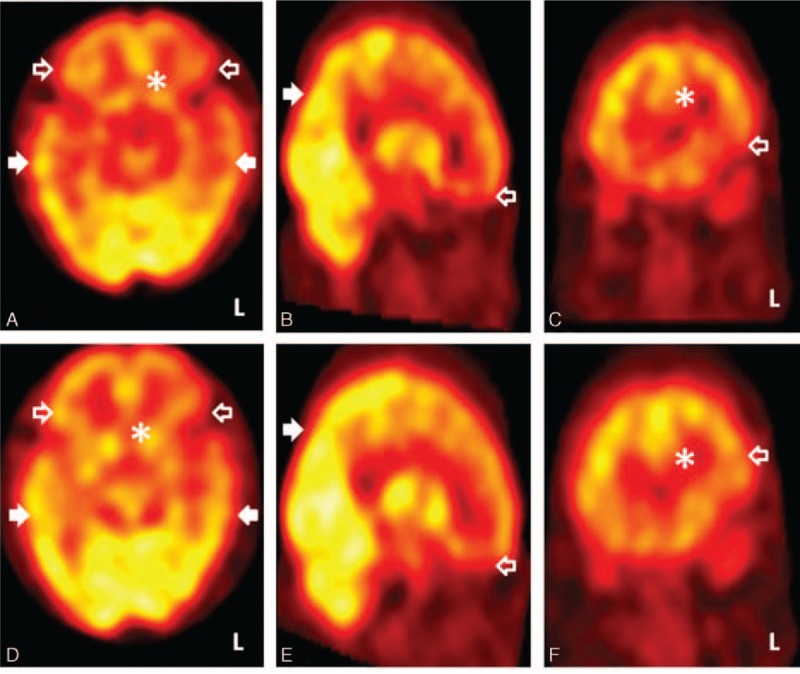
Tc-99m ethyl cysteinate dimer single-photon emission computed tomography of Case 1 (the poor responder) showing persistent hypoperfusion in the prefrontal cortex (hollow arrow heads) and anterior cingulate gyrus (asterisk) after cobalamin supplement therapy, in the presence of a certain degree of signal reversal in temporo-parieto-occipital regions (solid arrow heads). Upper row: before treatment; lower row: after treatment. (A) and (D): axial views, (B) and (E): sagittal views, (C) and (F): coronal views, color scale: window, 100; base, 0.

Case 1: A 74-year-old woman who had been a vegan for more than 10 years presented with a slowly progressive dull response and forgetfulness for 1 year. Except for a history of well-controlled hypertension, she had no history of diabetes mellitus, chronic kidney disease, atrial fibrillation, and cigarette consumption. Neurological examinations showed normal gait and deep tendon reflex throughout. Her serum Cbl level was 199 pg/mL (lower limit: 250 pg/mL) and homocysteine level was 19.14 μmol/L (upper limit: 17.2 μmol/L). Her serum low-density lipoprotein was 99 mg/dL (upper limit: 100 mg/dL); total cholesterol level was 167 mg/dL (upper limit: 200 mg/dL); glycated hemoglobin was 5.3% (upper limit: 5.6%); creatinine was 0.8 mg/dL (upper limit: 1.0 mg/dL); folic acid was 7.88 ng/mL (lower limit: 3.0 ng/mL). Other serology profiles relevant to depression and/or dementia including thyroid function and levels of cortisol were unremarkable. She scored 12/30 and 34/100 on the mini-mental state examination (MMSE) and cognitive abilities screening instrument (CASI), respectively, which were far below average (Table 1). Profound deficits in mental manipulation, drawing, short-term/long-term memory, and verbal fluency were noted. The total score of Neuropsychiatric Inventory was 2, resulted from a mild change in the subdomain of depression/dysphoria (frequency × severity = 2 × 1; mildly associated caregiver distress). Brain magnetic resonance imaging (MRI) showed only small periventricular caps (Fig. 1A) and mild senile atrophy (Fig. 1B); the magnetic resonance angiography showed patent major intracranial arteries. Tc-99m-ECD SPECT showed widespread hypoperfusion in bilateral frontotemporal regions (Fig. 2A). Cerebral perfusion within the anterior cingulate gyrus (ACC) (Fig. 2A and C) and prefrontal cortex (PFC) (Fig. 2A to C) was markedly impaired, presenting as ragged and discontinuous signals. Both dorsolateral and medial portions of the PFC were affected, and hypoperfusion within the bilateral thalamus and the left basal ganglia was also noted. She was diagnosed as having dementia due to Cbl deficiency. Her serum Cbl level normalized to 449 pg/mL 5 months after Cbl supplement therapy (cyanocobalamin 1500 ug/d). Although follow-up Tc-99m-ECD SPECT showed mild improvements in bilateral temporal cortices (Fig. 2D), signal recovery within the ACC (Fig. 2D and F) and PFC (Fig. 2D to F) was limited, consistent with no improvements in neuropsychological test scores (follow-up MMSE: 11/30, CASI: 39/100) (Table 1). At the last retest performed 1 year later, there was no further deterioration in her cognitive performance (MMSE: 12/30, CASI: 38/100). She did not take any antidepressants/acetylcholinesterase inhibitors but antihypertensives and Cbl supplement during the whole follow-up period.

**Table 1:**
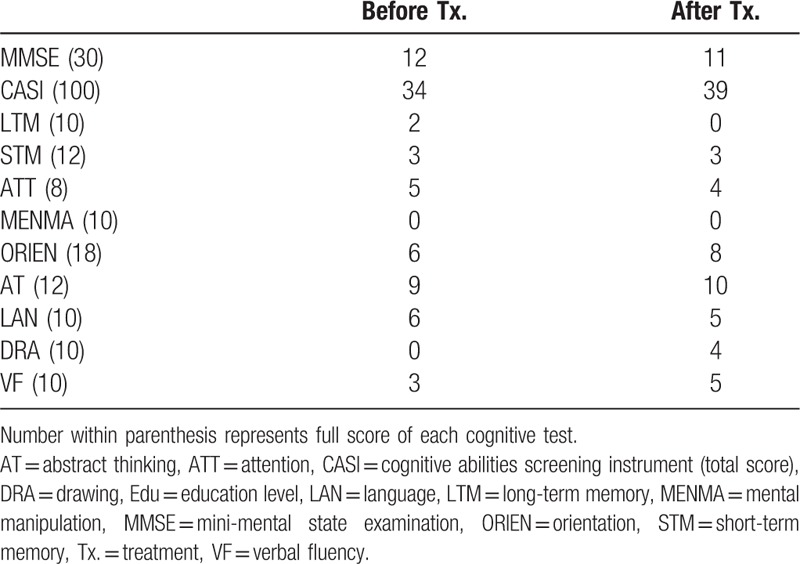
Cognitive performances of Case 1.

**Figure 3:**
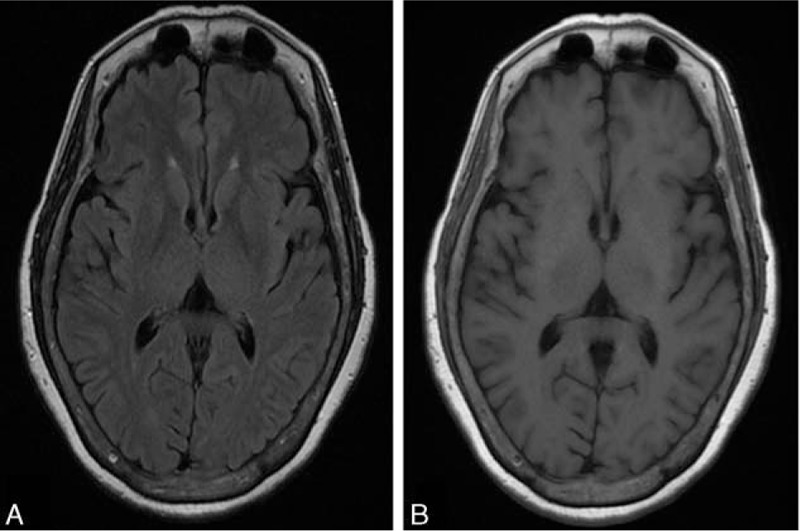
Brain magnetic resonance imaging of Case 2 showing normal appearing brain parenchyma, which was disproportionate to her cognitive deficits (A) T2 fluid-attenuated inversion recovery image, axial view. (B) T1-weighted image, axial view.

**Figure 4:**
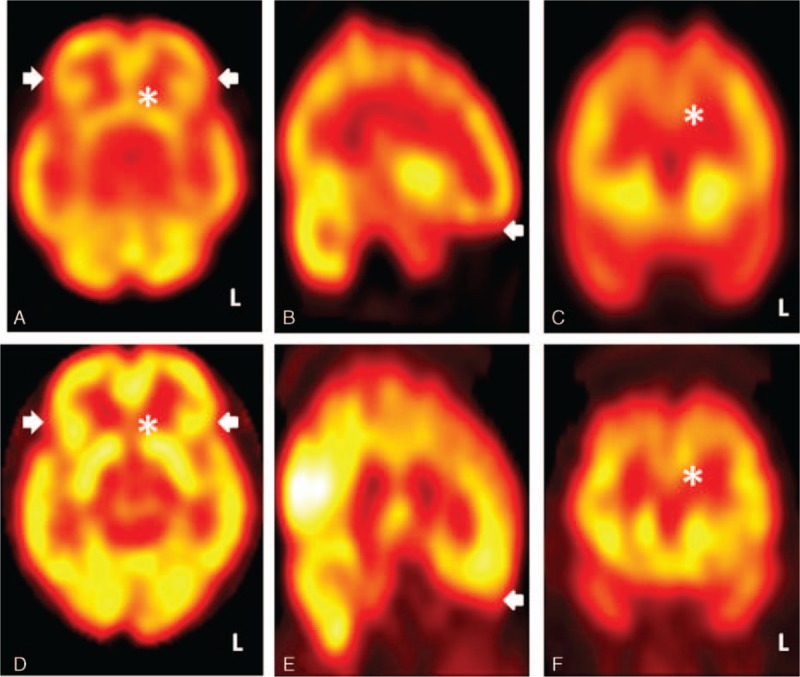
Tc-99m ethyl cysteinate dimer single-photon emission computed tomography of Case 2 (the good responder) showing global signal reversal, especially in the prefrontal cortex (solid arrow heads) and anterior cingulate gyrus (asterisk) after cobalamin supplement therapy. Upper row: before treatment; lower row: after treatment. (A) and (D): axial views, (B) and (E): sagittal views, (C) and (F): coronal views, color scale: window, 100; base, 0.

Case 2: A 61-year-old woman who had been a vegetarian for 5 years presented with insidious-onset of progressive memory complaints for 1 year. Aside from her cognitive complaints, she did not experience any sensation changes of limbs or gait problems. Except for age, she was free from risk factors of cerebrovascular disease that are commonly identified (e.g., hypertension, diabetes mellitus, chronic kidney disease, atrial fibrillation, and cigarette consumption). Her serum Cbl level was 207 pg/mL, in association with an elevation of homocysteine level (17.50 μmol/L; upper limit: 17.2 μmol/L). Laboratory data aiming on cerebrovascular risk assessment and cognitive deficits, including lipid profiles, glycated hemoglobin, creatinine, folic acid, thyroid function, and cortisol level, were all within reference ranges. She scored 24/30 on the MMSE and 78/100 on the CASI, mainly due to impaired mental manipulation and abstract thinking (Table 2). Borderline performance in short-term memory and verbal fluency were also noted. The total score of Neuropsychiatric Inventory was 2, resulted from a small change in the subdomain of sleep and nighttime behavior disorders (frequency × severity = 2 × 1; minimally associated caregiver distress). Tc-99m-ECD SPECT was performed due to the absence of obvious atrophy or vascular lesions in brain MRI (Fig. 3A and B). Although mildly impaired radio uptake was noted in bilateral frontal–temporal cortices (Fig. 4A), cerebral perfusion within the ACC (Fig. 4A and C) and PFC was relatively preserved (Fig. 4A and B). Additionally, there was hypoperfusion within the bilateral thalamus. She was diagnosed with mild cognitive impairment due to Cbl deficiency. Her Cbl level normalized (687 pg/mL) 5 months after the same Cbl supplement therapy as Case 1. Follow-up SPECT showed avid global improvements (Fig. 4D), including a remarkable signal reversal within the PFC (Fig. 4D and E) and ACC (Fig. 4D and F), consistent with favorable clinical recovery (follow-up MMSE: 28/30, CASI: 85/100) (Table 2). Of note, her initial deficits in short-term memory completely reversed.

**Table 2:**
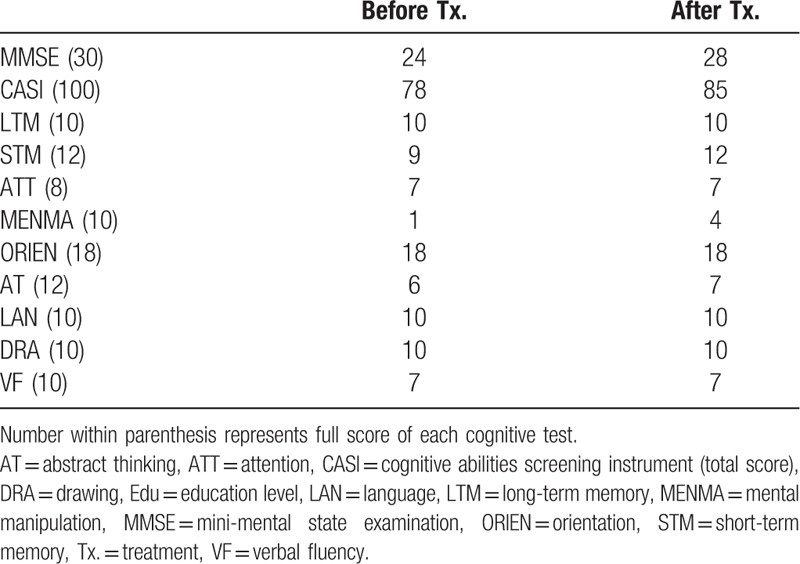
Cognitive performances of Case 2.

## 3 Discussion

The aim of these case reports is to describe and compare Tc-99m-ECD SPECT findings in 2 patients with Cbl deficiency but distinct therapeutic responses. The findings showed changes in cerebral perfusion along with cognitive performance, and localized signal reversal after supplement therapy. Differentiating Tc-99m-ECD SPECT patterns according to the therapeutic responses may assist in identifying potential biomarkers associated with prognosis and pathogenesis. Both patients had hypoperfusion within the frontal–temporal regions before treatment, and the extent and severity of this hypofrontality were likely responsible for the unfavorable prognosis. Specifically, radio uptake within both PFC and ACC may be considered to be a biomarker associated with therapeutic response. The principal activity of the PFC is orchestration of thought and action in accordance with internal goals.^[5]^ Its dynamic filtering mechanism, a prerequisite for executive function, has been implicated in goal-directed activations and irrelevant noise inhibition.^[6]^ Relevant widespread projections from the PFC are global and have a vital impact on cognition. The ACC is also considered to be a highly influential region for cognition, as it integrates neuronal connectivity of both frontosubcortical circuits and the limbic system.^[7]^ Therefore, advanced cognitive decline may develop as metabolic derangements of the ACC lead to dysfunction over the frontal-temporal regions through a mechanism of diaschisis. Our Tc-99m-ECD SPECT findings also highlight the role of the frontotemporal regions in cognition, as both of our cases showed impaired performance in memory and verbal fluency.

Cbl deficiency may contribute to cognitive decline through several mechanisms. First, symmetric and widespread hypometabolism within the frontal–temporal regions supports that Cbl deficiency impairs baseline cerebral metabolic tone. Given that Cbl plays a pivotal role in methylation and DNA synthesis,^[1]^ Cbl deficiency potentially compromises the integrity of cellular membranes and energy reserve of neurons. Moreover, monoamine neurotransmitter production may also be affected, as Cbl stimulates tetrahydrobiopterin synthesis, an important pathway for monoamine synthesis.^[1]^ Second, Cbl deficiency may impair cerebral perfusion via elevated levels of homocysteine, which has been reported to have a vasculotoxic effect.^[1,8]^ Although both cases showed hyperhomocysteinemia, the initial level of serum homocysteine in *Case 1* appeared to be higher than *Case 2*. In view of a priori knowledge related to homocysteine,^[9]^ the baseline serum level of homocysteine might possibly reflect the severity of its downstream oxidative stress. As *Case 1* still had progressive mental decline and limited signal reversal from the Tc-99m-ECD SPECT despite Cbl supplement, it seems that a relatively longer deficient state would cause irreversible degradation from the viewpoint of neuronal metabolism. In addition, the derangement of metabolic and microstructural changes may cause downstream dysfunction of ion channels and second messengers as well as neuronal membrane instability,^[1]^ contributing to profound cognitive deficits in the absence of structural changes in brain parenchyma.

Some may speculate the possibility that our patients had coexisting neurodegenerative diseases, such as Alzheimer disease or vascular dementia. However, we believe that such possibility was less likely. From a clinical viewpoint, the initial cognitive deficits of both cases went far beyond structural changes in brain MRI. The volumes of the medial temporal lobes were visually regarded to be nearly normal in terms of their age. In addition, both cases had minimal white matter changes in conventional brain MRI, which we presumed to have a limited impact on cognition. Although *Case 1* showed no apparent improvement after Cbl supplement therapy, a relatively stationary cognitive performance during long-term follow-up supported that her cognitive deficits were primarily due to low Cbl status. Furthermore, *Case 2* benefited remarkably from Cbl supplement therapy. The domain-specific improvement in short-term memory was very different from the therapeutic response expected in Alzheimer disease, where benefits from acetylcholinesterase inhibitors mainly result in attention, executive, language, and visuospatial function.^[10,11]^ From a neuroradiological viewpoint, the symmetric hypoperfusion within the frontal–temporal regions on SPECT represents a distinct pattern from Alzheimer disease-related pathology. Previous studies have validated that radio-uptake in the precuneus, posterior cingulate gyrus, and parietal and medial temporal lobes are most commonly impaired in patients with Alzheimer disease.^[12,13]^ Another recent SPECT report of mild cognitive impairment investigated the earlier stage and identified asymmetric hypoperfusion over the hippocampus in association with amnestic subtypes of mild cognitive impairment.^[14]^ Taken together, the Tc-99m-ECD SPECT findings in the current cases corroborate that the fundamental pathogenesis of Cbl deficiency-related cognitive decline is different from Alzheimer disease.

## 4 Conclusions

Our Tc-99m-ECD SPECT findings highlight the negative impact of Cbl deficiency on cerebral perfusion/metabolism. While symmetric hypoperfusion within the frontal–temporal regions was present in both cases, the severity of hypofrontality was correlated with cognitive performance before and after treatment. Signal integrity alongside the PFC and ACC may predict preservation of global cognitive function in patients with Cbl deficiency.

## Acknowledgments

The authors thank the patients and their caregivers for their time and commitment to this research. We also appreciate Mr. Tien-Hsin Chang, who assisted in SPECT imaging processing.
